# Redox homeostasis and inflammation in fibroblasts of patients with Friedreich Ataxia: a possible cross talk

**DOI:** 10.3389/fnmol.2025.1571402

**Published:** 2025-04-16

**Authors:** Andrea Quatrana, Sara Petrillo, Caterina Torda, Eleonora De Santis, Enrico Bertini, Fiorella Piemonte

**Affiliations:** ^1^Unit of Muscular and Neurodegenerative Diseases, Bambino Gesù Children’s Hospital, IRCCS, Rome, Italy; ^2^Research Unit of Neuromuscular Diseases, Bambino Gesù Children’s Hospital, IRCCS, Rome, Italy

**Keywords:** Freidreich Ataxia, Nrf2, NF-kB, TLR4, GLRX1, Trx1, IL-1β

## Abstract

Redox homeostasis is impaired in Friedreich’s Ataxia (FRDA), a neurodegenerative disease caused by the decreased expression of the mitochondrial protein frataxin. Nrf2, the master regulator of tissue redox balance, is defective in the disease, driving cells to ferroptosis. Neuro-inflammation is recently emerging as an additional pathological mechanism in FRDA and has to be understood in order to go deeper into the pathogenesis of the disease. As a functional cross talk between Nrf2 and NF-kB pathways has been previously reported, we wonder if inflammation may be activated in FRDA as a consequence of Nrf2 deficiency. Thus, we analyzed the expression of proteins involved in the antioxidant and inflammatory responses in fibroblasts of patients with FRDA. We found a significant activation of the TLR4/NF-kB/IL-1β axis in patients, associated to a consistent increase of the redox enzymes thioredoxin 1 (TRX1) and glutaredoxin 1 (GLRX1), which are essential to activate NF-kB under oxidative stress conditions. Furthermore, we investigated the role of 4-HNE, a by-product of lipid peroxidation, as a potential mediator between ferroptosis and inflammation in FRDA.

## 1 Introduction

Redox homeostasis is significantly altered in Friedreich’s Ataxia (FRDA; OMIM #229300), an inherited neurodegenerative disease caused by GAA repeat expansion within the first intron of the FXN gene coding for the mitochondrial protein frataxin (Fxn) (Keita et al., 2022; Krasilnikova et al., 2023).

FRDA is a chronic autosomal recessive cerebellar neurodegenerative condition with a prevalence of about 1:50.000 in Caucasians and a carrier frequency in the general population estimated at 1:100. Fxn is a highly conserved 210 amino acid mitochondrial protein ubiquitous across species. It plays a role in the mitochondrial biogenesis of Iron–Sulfur (Fe-S) clusters that contribute to various cellular functions, including redox catalysis, *β*-oxidation of lipids, regulation of gene expression, DNA repair/replication, and the proper functioning of oxidative phosphorylation (Monfort et al., 2022). Clinically, FRDA is characterized by slowly progressive ataxia, hypo−/areflexia, dysarthria, and peripheral neuropathy with loss of proprioceptive sensation. Symptoms usually manifest during childhood or adolescence, with loss of coordination, weakness, and fatigue, gradually leading patients to motor incapacity around 15–20 years after disease onset (Keita et al., 2022). The Fxn deficiency, primarily affecting dorsal root ganglia, cerebellum, and spinal cord neurons, perturbs iron homeostasis, causing mitochondrial impairment, overload of reactive oxygen species (ROS) and lipid peroxidation, ultimately triggering ferroptosis (Du et al., 2020; La Rosa et al., 2020a; Turchi et al., 2020).

Ferroptosis is an iron-dependent cell death caused by impaired glutathione metabolism, lipid peroxidation and mitochondrial failure. Emerging evidences report a role for ferroptosis in FRDA (Cotticelli et al., 2019; La Rosa et al., 2020b), with Nrf2 as main regulator of several genes directly or indirectly involved in modulating it (La Rosa et al., 2020b; Paupe et al., 2009; D’Oria et al., 2013; Shan et al., 2013; Petrillo et al., 2017, 2019, 2021; La Rosa et al., 2019, 2020b) and, importantly, the Nrf2 deficiency has been shown to activate the pathway of NF-κB, leading to increased production of inflammatory factors (Wardyn et al., 2015; Gao et al., 2022). Notably, the antioxidant enzymes glutaredoxin (GLRX) and thioredoxin (TRX) are able to interact with NF-kB, contributing to regulate the inflammatory cellular response (Seco-Cervera et al., 2020; Knoke and Leichert, 2023).

In this study, to explore a potential cross-linking between oxidative stress and inflammation in FRDA, we investigated the expression of GLRX1, TRX1, NF-kB, and IL-1β in fibroblasts of three patients with FRDA, in order to highlight whether the redox imbalance might activate some inflammatory response in patients. Furthermore, as the Toll-like receptor 4 (TLR4) is regulated by GLRX1 (Chantzoura et al., 2010; Moghimpour Bijani et al., 2012) and we recently found, by comparative transcriptomic analysis, a significant increase of TLR4 in one of two sisters both displaying fxn deficiency but only one of them symptomatic (Petrillo et al., 2024), we also measured the TLR4 content in FRDA fibroblasts, to investigate its possible involvement in the disease.

Finally, since TLR4 is modulated by ferroptosis and it is activated by the ferroptosis hallmark 4-hydroxynonenal (4-HNE) (Gargiulo et al., 2015; Hsu et al., 2022), and we previously found increased plasma levels of 4-HNE in patients (La Rosa et al., 2020b), we further analyzed the 4-HNE content in FRDA fibroblasts, to look for a potential mediator between ferroptosis and inflammation in this disease.

## 2 Materials and methods

### 2.1 Fibroblasts cultures and treatments

Skin biopsies were taken from three clinically affected (and genetically proven) FRDA patients (Table 1) and three age-matched healthy subjects (Ctrls). Fibroblasts were grown in Dulbecco’s modified Eagle’s medium supplemented with 10% fetal bovine serum, 50 units/mL penicillin, 50 μg/mL streptomycin 0.4% (v/v), at 37°C (5% CO2). Fibroblasts, cultured to 70% confluence, were used at similar passage numbers (9–11). Ferroptosis was induced by incubating cells for 3 h with 250 nM RSL3 (Zhang et al., 2018). After washing, cells were lysed in Total RNA Purification Plus Kit (Norgen Biotek Corp., Torold, ON, Canada), according to the manufacturer’s protocol for RNA extraction and subjected to quantitative Real-Time PCR or lysed with RIPA buffer including DTT and protease inhibitors for Western blotting analysis. Cells were used at similar, 9–11, passage numbers and tested for mycoplasma contamination. All the participants signed an informed consent and the study was approved by the Ethics Committee of “Bambino Gesù” Children’s Hospital (code 1166/2016; date of approval 08/06/2016).

**Table 1:** Clinical data of patients with FRDA.

Patient	Age (yrs)	Sex	GAA repeats	Cardiomyopathy	Diabetes
#1	19	F	680/350	No	No
#2	14	M	848/848	Yes	No
#3	8	M	448/848	Yes	No

### 2.2 Western blot analysis

Fibroblasts (1×10^6^) were lysed on ice with RIPA buffer and protease inhibitors. An amount of 30 μg proteins was subjected to SDS PAGE on 4–12% denaturing gel and probed with the following antibodies: NF-kB p65 (1:1000, 14–6,731-81; Invitrogen, USA), IL-1 beta (1:1000, P420B; Invitrogen, USA), Glutaredoxin-1/GLRX1 (1:1000, NBP2-55346; Novus Biologicals, Bio-Techne, USA), Thioredoxin-1/TRX1 (1:1,000, MAB19701; Novus Biologicals, Bio-Techne, USA), Nrf2 (1:500, ab31163; Abcam, UK), and GAPDH (1:10,000, #G9545; Sigma Aldrich) as loading control. Immunoreactive bands were detected using the Lite Ablot Extend Long Lasting Chemiluminescent substrate (Euroclone, Milan, Italy). Signals derived from appropriate HRP-conjugated secondary antibodies (Bethyl Laboratories, Montgomery, TX, United States) were captured by Chemi DocTM XRS 2015 (Bio-Rad Laboratories, Hercules, CA, United States) and densitometric analysis was performed using Image Lab software (Version 5.2.1, Bio- Rad Laboratories).

### 2.3 Quantitative real time PCR (qRT-PCR)

One μg of total RNA per sample was reverse transcribed with the SuperScript. First-Strand Synthesis system and random hexamers as primers (Life Technologies, Carlsbad, CA, United States). The expression levels of NF-kB, IL-1β, TLR4, TRX1, and GLRX1 were measured by qRT-PCR in an ABI PRISM 7,500 Sequence Detection System (Life Technologies) using Power SYBR Green I dye chemistry (ThermoFisher Scientific, Walthman, MA, United States). Data were analyzed using the 2-^ΔΔCt^ method with TBP (TATA box binding protein) and Glyceraldehyde-3-phosphate dehydrogenase (GAPDH) as housekeeping genes. Data are shown as fold change relative to controls. Primers used for qRT-PCR are reported in Table 2. mRNA expression of the TLR4 gene was normalized to the TATA-box-binding protein, TBP, using the TaqMan^®^ Gene Expression Master Mix, with the following TaqMan^®^ Gene Expression Assays primers and probes (Applied Biosystems): TLR4, Hs00152939_m1 and TBP, Hs00427620_m1.

**Table 2:** Primer sequences used for qRT-PCR.

Gene	Forward	Reverse
GLRX1	CGATATCACAGCCACCAACCAC	GACTCGAGGCACCGTTCTTG
TRX1	GAAGGGACAAAAGGTGGGTGA	ATGGCAACTGGGTTTATGTCTTCA
NF-kB p65	CGCTGCATCCACAGTTTCCAGA	AGTCCCCACGCTGCTCTTCTAT
TBP	CCGAAACGCCGAATATAAT	AAATCAGTGCCGTGGTTCGT

GLRX1, Glutaredoxin-1; TRX1, Thioredoxin-1; NF-kB p65, Nuclear factor-κB p65 subunit; TBP, TATA box binding protein.

### 2.4 4-HNE assay

The quantitative measurement of 4-HNE on FRDA fibroblasts was performed by a competitive ELISA kit (Lipid Peroxidation 4-HNE Assay kit, Abcam, Cambridge, UK), which allows the quantitation of the 4-HNE adduct by comparing its absorbance with that of a known 4-HNE-BSA standard curve. Cells were homogenized in RIPA lysis buffer, centrifuged at 12,000 × g for 10 min, and the supernatant was collected. Samples absorbance was detected on a microplate reader (Enspire, PerkinElmer, USA) at 450 nm. Protein concentration was detected by the BCA method (ThermoFisher, Walthman, MA, United States) and 4-HNE levels were normalized to protein concentrations of each sample.

### 2.5 IL-1β detection by ELISA

Cultured FRDA and control fibroblasts were collected and re-suspended in cold RIPA buffer, then subjected to sonication till the suspension was clarified and centrifuged at 1,500 × g for 10 min at 4°C to remove cellular debris. IL-1β concentrations were measured using the enzyme-linked immunosorbent assay Human IL-1 beta ELISA Kit (RAB0273, Sigma-Aldrich) on a microplate reader (Enspire, PerkinElmer, USA) at 450 nm and quantified using a standard curve, according to the manufacturers’ instructions. Cytokine levels were normalized to protein concentrations of each sample.

### 2.6 Statistical analysis

Statistical analysis was performed using the GraphPad Prism 5.0 Software (San Diego, CA, United States). Statistically significant differences between two groups were analyzed using Student’s *t*-test and comparisons between multiple groups by one-way ANOVA. All data are shown as mean ± SEM. Statistical significance was defined as *p < 0.05, **p < 0.001, ***p < 0.001 and ****p < 0.0001 compared to healthy controls or to untreated samples to compare each treatment condition.

## 3 Results

### 3.1 NF-kB and IL-1β are increased in fibroblasts of patients with FRDA

**Figure 1:**
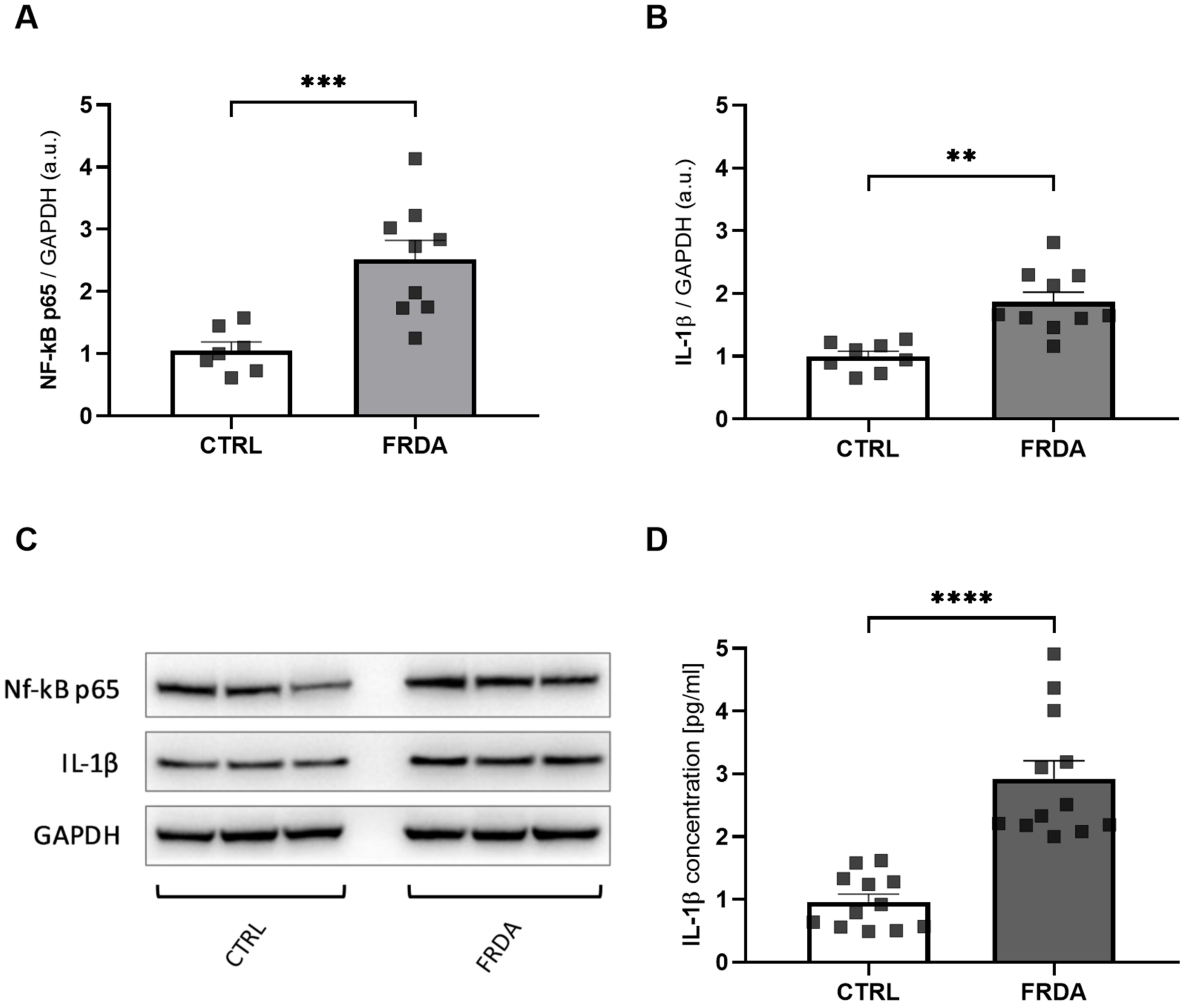
Densitometry of NF-kB p65 **(A)** and IL-1β **(B)** protein levels in fibroblasts of n.3 patients with FRDA vs. n.3 control cells (CTRLs), as determined by Western blot analysis. **(C)** Representative Western blot images of NF-kB p65, IL-1β and GAPDH. **(D)** IL-1β content as measured by ELISA in FRDA cells. Values are expressed as mean ± SEM. Statistical significance was determined by Student’s *t* test and defined as *p < 0.05, **p < 0.01, ***p < 0.001 and ****p < 0.0001 respect to controls.

Starting from the negative cross-talk between the two main regulators of oxidative stress and inflammation (Wardyn et al., 2015; Gao et al., 2022; van der Horst et al., 2022), we analyzed levels of NF-kB and IL-1β in fibroblasts of n.3 FRDA patients and n.3 healthy subjects, to understand if the frataxin-mediated Nrf2 decrease might be followed by an inflammatory response in FRDA. As shown in Figures 1A,C we found a significant increase of NF-kB expression in patients, along with a consistent rise of IL-1β (Figures 1B,C). The quantification by ELISA confirmed the increase of IL-1β in FRDA fibroblasts (Figure 1D).

### 3.2 GLRX1 and TRX1 are up-regulated in FRDA fibroblasts

**Figure 2:**
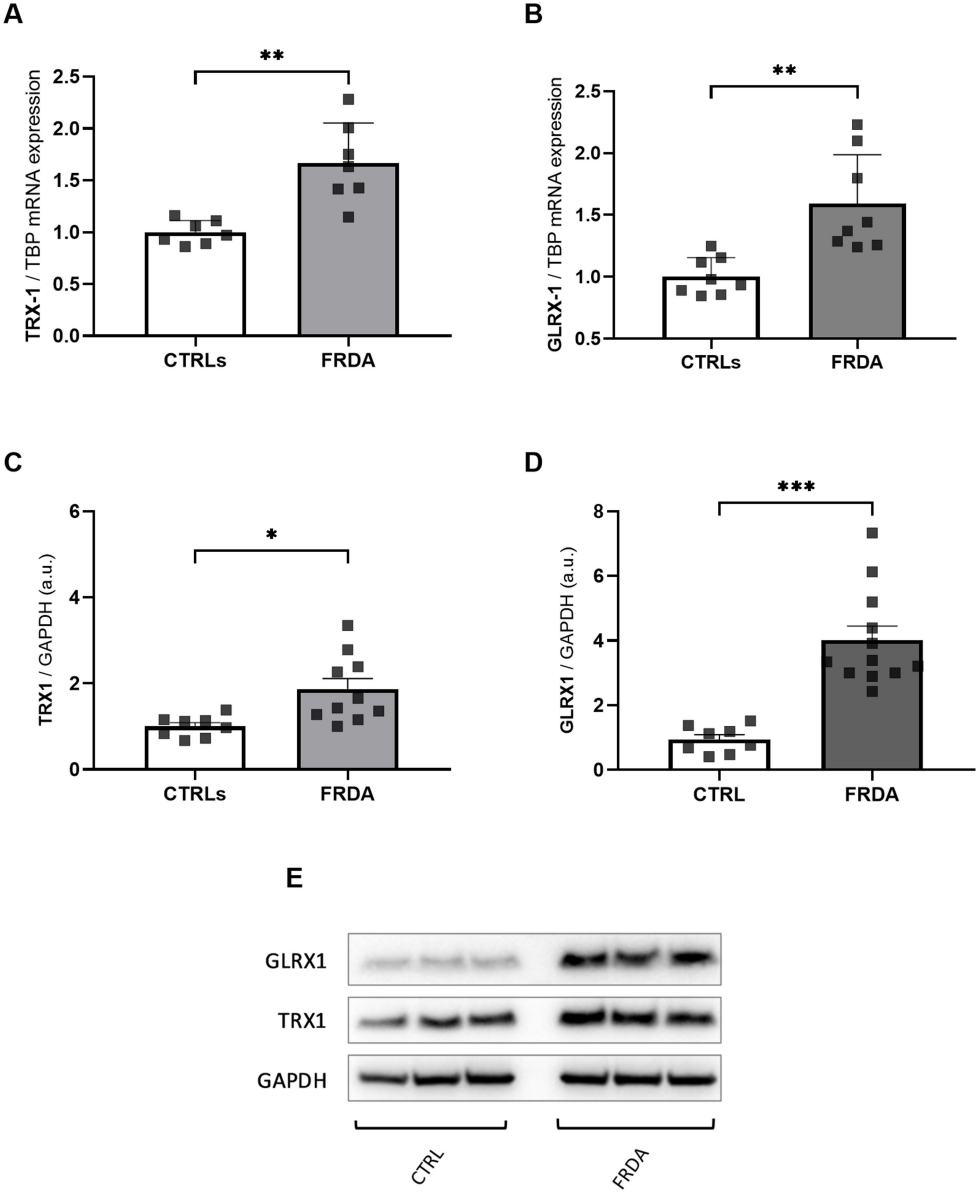
mRNA **(A,B)** and protein **(C,D)** levels of TRX1 and GLRX1 in n.3 FRDA fibroblasts and n.3 control subjects, as determined by qRT-PCR and Western blot analysis, respectively. **(E)** Representative Western blot of TRX1, GLRX1, and GAPDH (as loading control). Data are expressed as mean ± SEM. Statistical significance was determined by Student’s *t* test and defined as *p < 0.05, **p < 0.01, ***p < 0.001 respect to controls.

NF-kB undergoes redox regulation in cells by the interaction with the antioxidant enzymes TRX1 and GLRX1 (Reynaert et al., 2006). In particular, NF-kB is negatively regulated by S-glutathionylation, and the action of GLRX1 overcomes this inhibition by catalyzing the de-glutathionylation of NF-kB and promoting its activation (Aesif et al., 2011). Also TRX1 contributes to keep NF-kB in an active state by reducing the disulfide bond of Cys62 in the p50 NF-kB subunit (Yoshioka et al., 2006). Thus, given the increase of NF-kB expression in FRDA fibroblasts, we asked if TRX1 and GLRX1 might be implicated in its up-regulation. As shown in Figure 2, significant increases of both enzymes have been observed in FRDA cells, when compared to healthy subjects, either as mRNA transcript levels (Figures 2A,B) and as protein amounts (Figures 2C–E), thus suggesting a dual role for TRX1 and GLRX1 in FRDA, as modulators of redox homeostasis but also as possible contributors in the inflammatory response.

### 3.3 TLR4 is increased in FRDA fibroblasts

**Figure 3:**
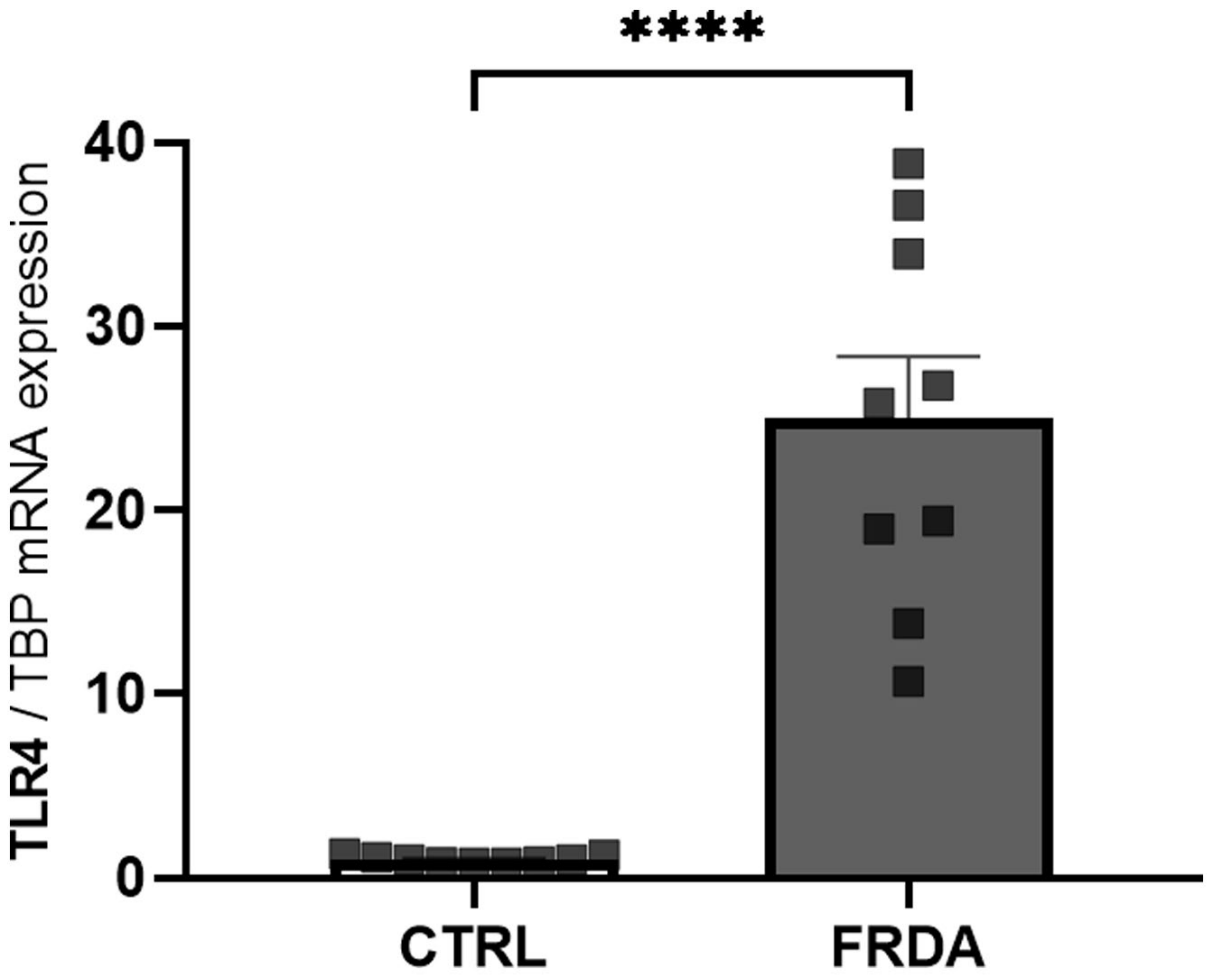
qRT-PCR analysis of toll-like receptor 4 (TLR4) expression in n. 3 FRDA fibroblasts and n. 3 controls (CTRLs). Values are expressed as mean ± SEM. Statistical significance was determined by Student’s *t* test and defined as ****p < 0.0001 respect to controls.

NF-kB is the effector of the signaling pathway activated by TLR4 (Baichwal and Baeuerle, 1997; Asehnoune et al., 2004), and the TLR4 function depends on the activity of GLRX1 (Chantzoura et al., 2010; Moghimpour Bijani et al., 2012). Therefore, given the increased expression of both NF-kB and GLRX1 in FRDA fibroblasts and moving from our recent evidence of a different TLR4 modulation in two sisters with FRDA (Petrillo et al., 2024), we wonder if TLR4 could be up-regulated in FRDA fibroblasts. As shown in Figure 3, we found a consistent increase of TLR4 mRNA levels in FRDA cells, respect to controls. This confirms the result obtained by comparative transcriptomic analysis on a family with FRDA (Petrillo et al., 2024), and support a role for TLR4 in the redox-mediated inflammation in this disease.

### 3.4 Ferroptosis induced the inflammatory response in control cells

**Figure 4:**
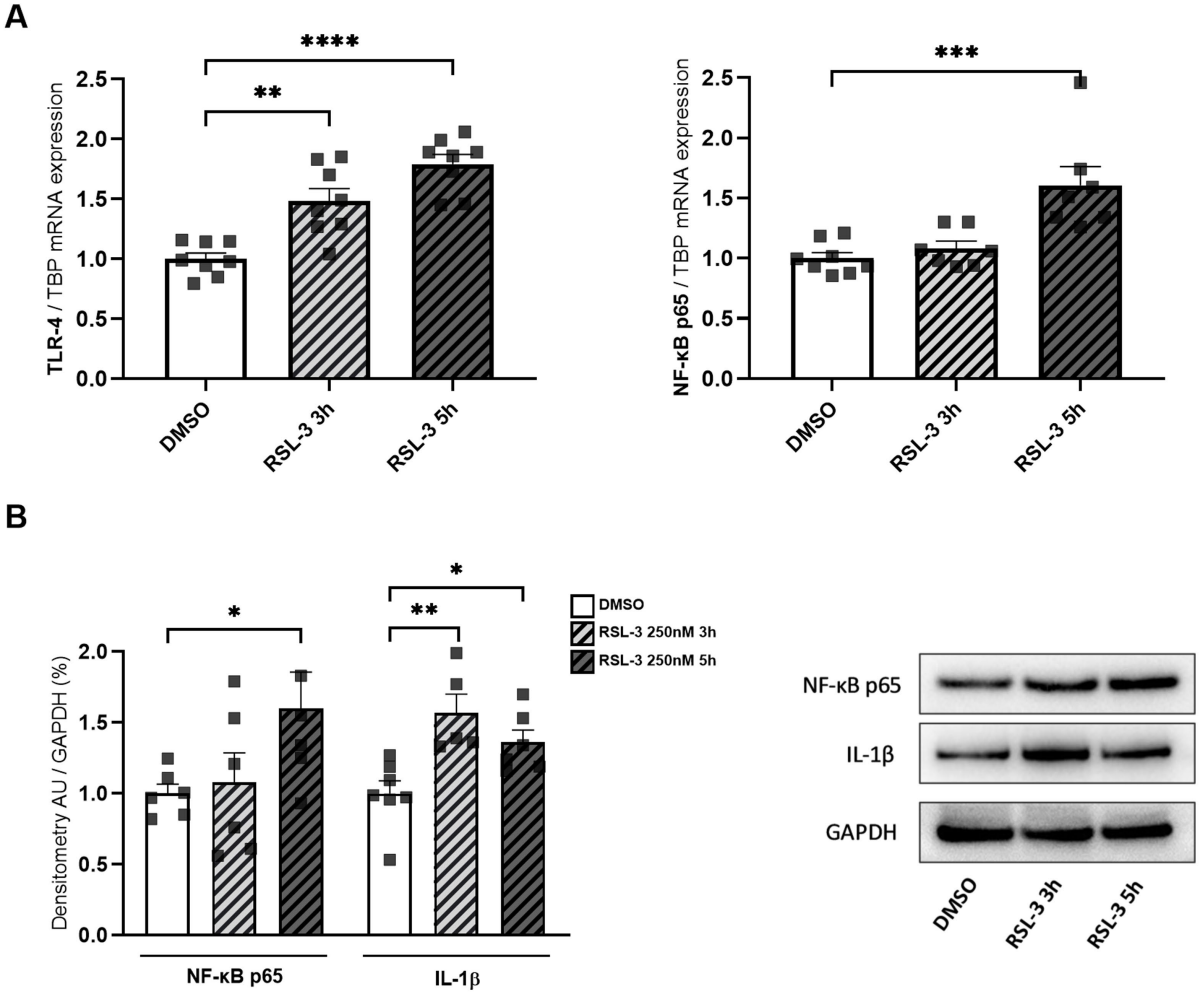
**(A)** TLR4 and NF-kB p65 mRNA expression upon RSL-3 treatment, as determined by qRT-PCR. **(B)** Western blot analysis of NF-kB p65 and IL-1β protein amounts, along with representative Western blot images of the respective proteins. Values are expressed as mean ± SEM. Statistical analyses were performed by ANOVA and significance was defined as *p < 0.05, **p < 0.01, ***p < 0.001 and ****p < 0.0001 respect to controls.

**Figure 5:**
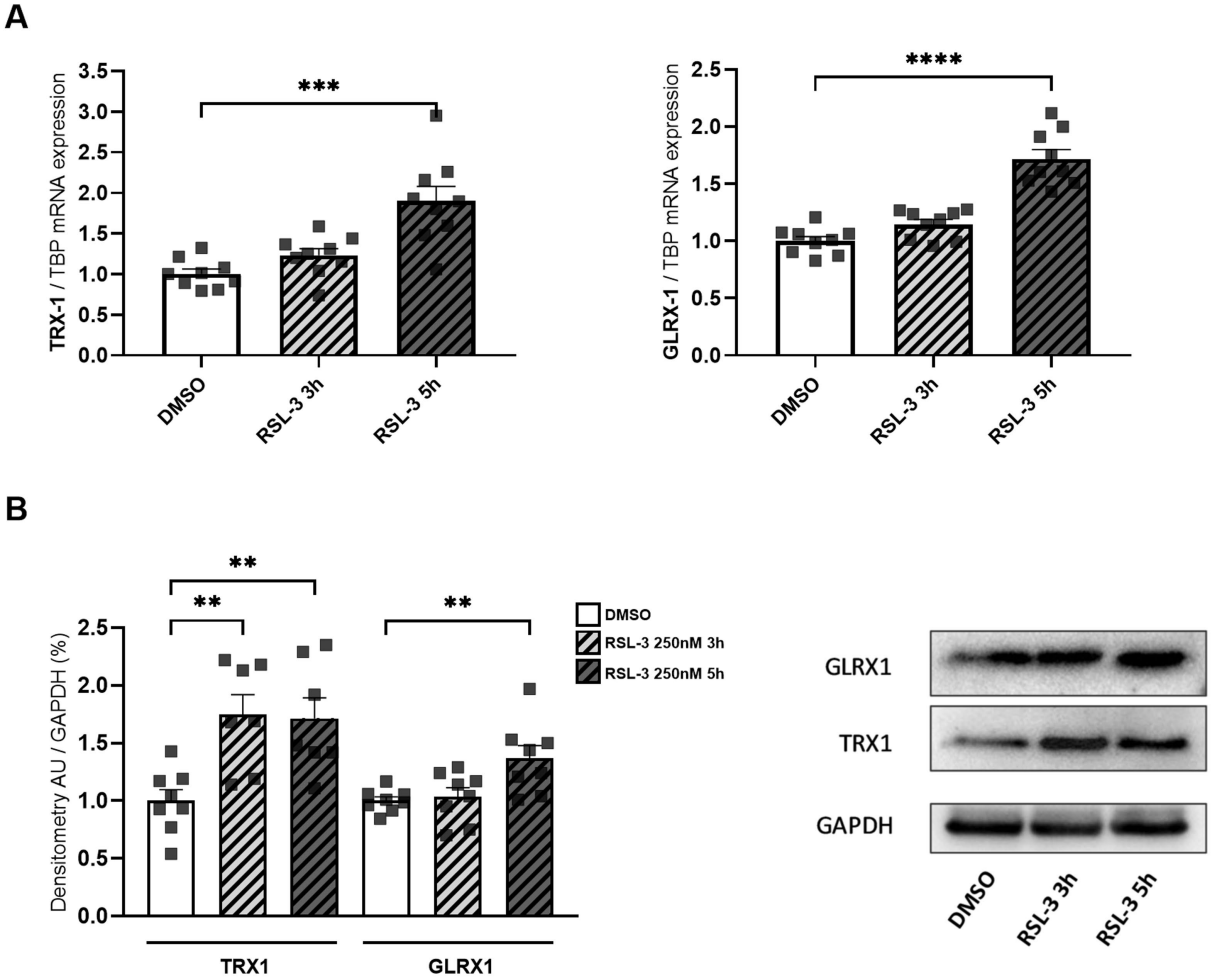
mRNA **(A)** and protein levels **(B)** of TRX1 and GLRX1 in control fibroblasts after RSL-3 treatment. A representative Western blot of TRX1 and GLRX1 is also reported. Values are expressed as mean ± SEM. Statistical analyses were performed by ANOVA and significance was defined as *p < 0.05, **p < 0.01, ***p < 0.001 and ****p < 0.0001 respect to controls.

Neuro-inflammation and ferroptosis are emerging as co-regulated mechanisms in the Disorders of Central Nervous System, and ferroptosis underlies pathogenesis in FRDA (La Rosa et al., 2020a; Sanz-Alcázar et al., 2024; He et al., 2024). Thus, we asked whether inducing ferroptosis in control cells could modulate the expression of NF-kB, IL-1β and TLR4. Therefore, we treated fibroblasts of healthy subjects with RSL-3, a well-known ferroptosis inducer, and we found increased levels of TLR4 and NF-kB mRNA following the treatment (Figure 4A). Western blot analysis (Figure 4B) confirmed the up-regulation of NF-kB and further evidenced a 50% induction of IL-1β protein expression. Interestingly, the RSL-3 treatment was able to increase also TRX1 and GLRX1 (Figure 5), either as mRNA levels (Figure 5A) or protein amounts (Figure 5B). These findings demonstrate a mutual regulation between ferroptosis and inflammation and suggest a role for TRX1 and GLRX1 at the crossroads of these two processes.

### 3.5 4-HNE: a potential mediator between ferroptosis and inflammation?

**Figure 6:**
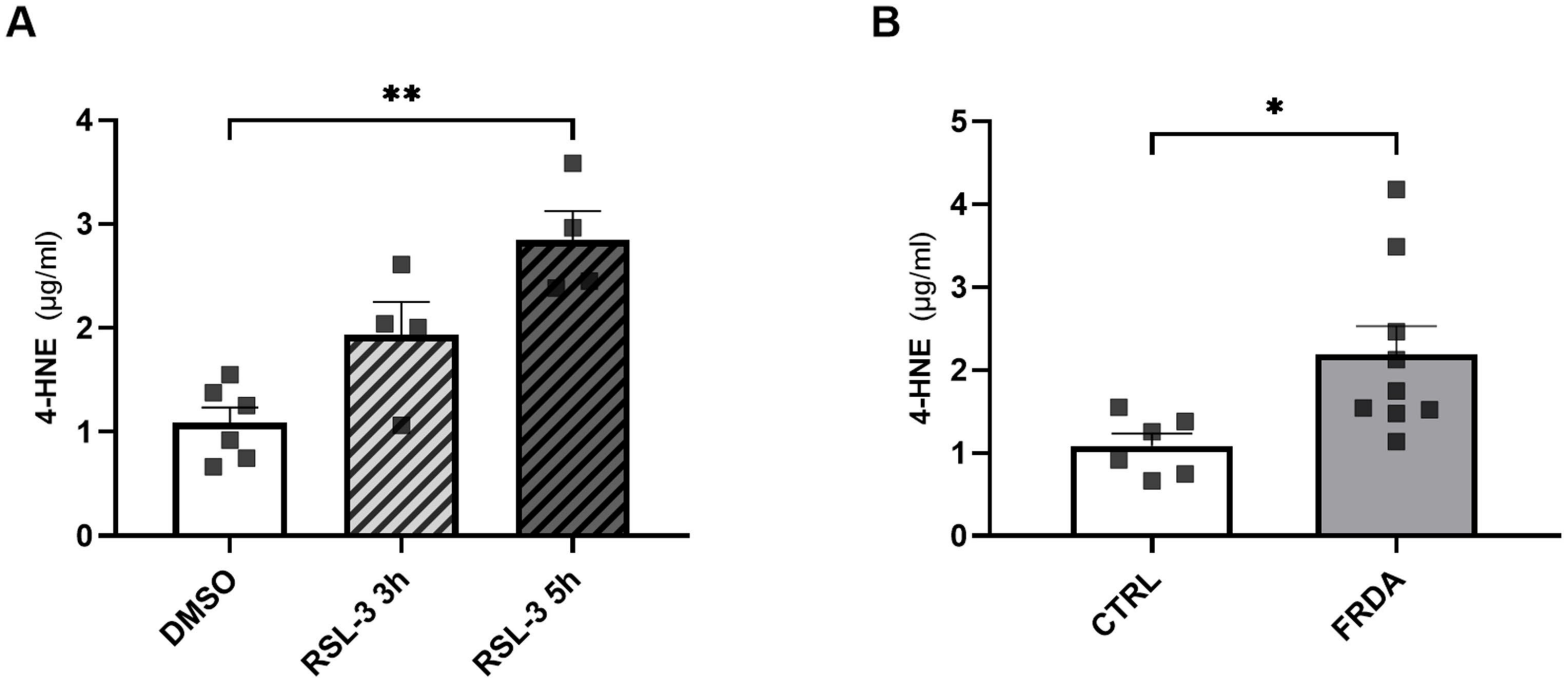
4-HNE levels, as measured by ELISA, in n.3 control fibroblasts incubated for 3 h and 5 h with 250 nM RSL3 **(A)** and in FRDA fibroblasts from n.3 patients **(B)**. Cells were used at similar passage numbers (9–11) and the assays performed in triplicates. Values are expressed as mean ± SEM. Statistical significance was determined by Student’s t test and defined as *p < 0.05 and **p < 0.01.

4-HNE, the most representative hallmark of ferroptosis (Mortensen et al., 2023), has been previously reported to modulate the activity of TLR4 and NF-kB signaling pathways (Gargiulo et al., 2015; Wang et al., 2019; Sharma et al., 2022). Thus, to explore a potential role for this by-product of lipid peroxidation in mediating ferroptosis and inflammation in FRDA, we analyzed the content of 4-HNE either in RSL-3-treated control cells and in fibroblasts of patients with FRDA. As reported in Figure 6, we found a progressive time-dependent rise of the 4-HNE content in control cells after ferroptosis induction (Figure 6A) and, importantly, we highlight a 2-fold increase of 4-HNE levels in fibroblasts of patients (Figure 6B). These findings, besides confirming ferroptosis in the pathogenesis of FRDA, may provide the basis for investigating 4-HNE as a potential mediator in the ferroptosis-driven inflammatory response in the disease.

## 4 Discussion

Oxidative stress represents a major pathophysiological contributor to FRDA onset and progression, and a great effort has been dedicated to restore redox balance in this disease (Lynch et al., 2024; Pilotto et al., 2024; Wang et al., 2024). However, the antioxidant therapies in clinical trials only partly reflect the promising results obtained in preclinical studies, indicating that additional pathways, potentially correlated to the antioxidant response, may be involved.

The down regulation of Nrf2 has been shown to modulate the pathway of NF-kB in Nrf2 deficient mice and in primary cultured astrocytes (Paupe et al., 2009; D’Oria et al., 2013; Shan et al., 2013; Wardyn et al., 2015; Petrillo et al., 2017, 2019, 2021; La Rosa et al., 2019, 2020b; Gao et al., 2022). Notably, the Nrf2 gene has a binding site for NF-kB (Lee et al., 2009; Jiang et al., 2013) and Nrf2 deficiency induces the degradation of IkBα, increasing NF-κB levels and triggering inflammation (Cuadrado et al., 2014). In particular, as reported by Gao et al. (2022), in the classical NF-kB pathway the activation of the IκK complex induced the phosphorylation of IκBα thus promoting its degradation and the NF-kB activation. Furthermore, using a dominant-negative mutant of IkBα, in which Ser32 and Ser36 had been changed to Ala, the IkB degradation was inhibited, demonstrating a new mechanism of RAC1 modulated inflammatory pathway through the IkBα-mediated cross-talk between NF-kB and NRF2.

Moving from those previous evidences showing a functional cross talk between Nrf2 and NF-kB pathways, in this study we analyzed the TLR4/NF-kB/IL-1β axis in fibroblasts of patients with FRDA, in order to understand if it could be modulated as a consequence of Nrf2 deficiency. The expression of NF-kB, together with its upstream signaling molecule TLR4 and the inflammatory cytokine IL-1β, were all significantly increased in fibroblasts of patients with FRDA, indicating a reactive inflammatory response in the disease. Furthermore, as NF-kB and TLR4 are negatively regulated by the oxidation, specifically via S-glutathionylation, and given that their inhibition is overcome by the action of the de-glutathionylating enzyme GLRX1 (Reynaert et al., 2006; Chantzoura et al., 2010; Aesif et al., 2011), we also analyzed the cellular content of the redox enzymes GLRX1 and TRX1 in FRDA fibroblasts, to understand if a correlation between redox homeostasis and NF-kB signaling pathway might occur in this disease. To note, NF-kB binding sites have been identified within the promoter of GLRX1 (Aesif et al., 2011), thus reinforcing the hypothesis of a coordinated GLRX1–mediated regulation of NF-kB.

The TRXs and GLRXs are a family of ubiquitous redox proteins, distributed among extracellular fluid, cytoplasm, mitochondria and nucleus, displaying antioxidant functions (Lu and Holmgren, 2012; Mahmood et al., 2013). Neuroprotective roles for these proteins are emerging in several neurodegenerative diseases (i.e., Alzheimer’s Disease, Parkinson’s Disease, Amyotrophic Lateral Sclerosis) (Johnson et al., 2015; McBean et al., 2015; Álvarez-Zaldiernas et al., 2016; Jia et al., 2021), although few studies have analyzed their involvement in FRDA (Shan et al., 2013; Seco-Cervera et al., 2020). TRX1 and GLRX1 were down-regulated in dorsal root ganglia, but up-regulated in nerve roots, of a FRDA mouse model (YG8R mice) (Shan et al., 2013) and discordant results have been reported in human fibroblasts (Seco-Cervera et al., 2020). Our findings show a consistent increase of GLRX1 and TRX1 in FRDA fibroblasts, thus opening the way for a dual role of these enzymes in FRDA, both as modulators of redox homeostasis but also as potential activators of the inflammatory response.

Specific pathways related to neuro-inflammation are altered in the microglia, astrocytes, and myelinating glial cells in FRDA (Lu et al., 2009; Apolloni et al., 2022; Imbault et al., 2022). In particular, using the brain positron emission tomography analysis, the authors revealed increased glial activation in the brain regions implicated in FRDA neuropathology, i.e., dentate nuclei, brainstem, superior cerebellar peduncles, and cerebellar cortex, compared to the control subjects. This was correlated with earlier disease onset, shorter disease duration, and the rise of some plasma inflammatory cytokines (i.e., IL-6), indicating chronic neuro-inflammation in the disease (Khan et al., 2022). Moreover, astrocytes lacking fxn displayed abnormal secretion of molecules associated with immunity and inflammation, and macrophage inflammatory protein-1 alpha (MIP-1*α*) (Khodagholi et al., 2018). The activation of immune system was among the earliest pathways regulated in the Fxn knockdown model (Chandran et al., 2017), and a strong activation of IL-6, IL-1β and TNF-α have been reported in human frataxin-deficient Schwann cell lines by microarray analysis (Lu et al., 2009). Altered patterns of proteins involved in the immune response have been reported in peripheral cells of patients and in mouse models by proteomic and transcriptomic analyses (Kulic and Unschuld, 2016; Imbault et al., 2022), and neuro-inflammation was also documented in DRGs, in the dentate nucleus, cerebellum and brainstem of FRDA patients (Koeppen et al., 2012, 2016; Khan et al., 2022).

Overall, these previous evidences support the importance to investigate inflammation in FRDA, not only to provide new insights into the knowledge of the disease pathogenesis, but also aimed at developing novel therapeutic approaches in this neurodegenerative disease. Furthermore, it must be considered that inflammation and ferroptosis are closely inter-connected (He et al., 2024) and, importantly, 4-HNE, the main ferroptosis hallmark, has been shown to play a critical role in the function of TLR4 (Kim et al., 2009; Wang et al., 2019). Therefore, the systemic increase of 4-HNE that we previously reported in blood of patients with FRDA (La Rosa et al. 2020b), together with the high 4-HNE levels in FRDA fibroblasts and its time-dependent increase following the ferroptosis induction, strongly support the hypothesis of 4-HNE as a potential mediator between ferroptosis and inflammation in FRDA.

In conclusion, although many questions remain open and more extensive analyses will be essential to confirm these data and translate them into therapeutic effective options, nevertheless our findings open the way to a new perspective in FRDA, with the Nrf2-mediated redox imbalance predisposing cells to the inflammation, thus contributing to exacerbate the disease activity. This study will provide new insights, particularly aimed at the development of synergic anti-inflammatory and antioxidant combined therapies.

## Data Availability

The original contributions presented in the study are included in the article, further inquiries can be directed to the corresponding author.
